# Adverse reactions to fluoroquinolones in the Nigerian population: an audit of reports submitted to the National Pharmacovigilance Centre from 2004 to 2016

**DOI:** 10.1002/prp2.297

**Published:** 2017-02-14

**Authors:** Ibrahim A. Oreagba, Kazeem A. Oshikoya, Comfort Ogar, Abiodun O. Adefurin, Ali Ibrahim, Olufunsho Awodele, Yetunde Oni

**Affiliations:** ^1^ Pharmacology, Therapeutics and Toxicology DepartmentCollege of MedicineUniversity of LagosIdiarabaLagosNigeria; ^2^ Pharmacology DepartmentLagos State University College of MedicineIkejaLagosNigeria; ^3^ National Pharmacovigilance CentreNational Agency for Food and Drug Administration and ControlAbujaNigeria; ^4^ Department of Internal MedicineMeharry Medical College1005 Dr. D.B. Todd Jr. Blvd.NashvilleTennessee

**Keywords:** Adverse reactions, database, fluoroquinolones, Nigeria, pharmacovigilance, spontaneous reports

## Abstract

Adverse drug reactions (ADRs) recorded in national pharmacovigilance databases in developed countries have been analyzed. However, adverse reactions to fluoroquinolones were observed globally despite their wide use and safety concerns. We provided information on the pattern of adverse reactions to fluoroquinolones reported spontaneously to the National Pharmacovigilance Centre (NPC), Nigeria. ADRs to fluoroquinolones reported to the NPC, over a period of 12 years, were analyzed. Evaluation was done for annual reports, age and gender of patients, type of reporter, suspected fluoroquinolones and adverse reactions, onset and outcome of ADRs, and causality. A total of 18527 ADR reports were received by the NPC. Antibiotics accounted for 1371(7.4%) of the total reports and fluoroquinolones accounted for 256 (18.7%) cases. A total of 540 ADRs due to fluoroquinolones was experienced by the patients. Multiple ADRs were experienced by 165 (65%) patients. Norfloxacin (2; 0.8%), moxifloxacin (3; 1.2%), ofloxacin (10; 3.9%), ciprofloxacin (112; 43.8%), and levofloxacin (129; 50.4%) were responsible for the ADRs. Neurological disorders (121; 22.4%), gastrointestinal disorders (118; 21.9%), and skin‐appendage disorders (116; 21.5%) were the most reported ADRs, while pruritus (41; 7.6%), abdominal pain (34; 6.3%), vomiting (34; 6.3%), and skin rash (27; 5.0%) were the most frequently reported specific ADRs. Thirty‐four (6.4%) patients experienced serious ADRs. Fluoroquinolones accounted for a small but significant proportion of ADRs spontaneously reported to the NPC in Nigeria. Ciprofloxacin and levofloxacin were the two most culpable fluoroquinolones due to their inappropriate use or increased use in multi‐drug resistant tuberculosis (MDR‐TB) treatment.

**Abbreviations**
ABECB: acute bacterial exacerbation of chronic bronchitis
ABS: acute bacterial sinusitis
ADR: adverse drug reaction
ART: adverse reaction terminology
CNS: central nervous system
EMEA: European Medicines Agency
GIT: gastro‐intestinal tract
ICSR: individual case study report
MDR‐TB: multi‐drug resistant tuberculosis
NAFDAC: National Agency for Food Drug Administration and Control
NPC: National Pharmacovigilance Centre
NPCs: National Pharmacovigilance Centres
SAERS: Saudi Adverse Event Reporting System
SJS: Stevens Johnson syndrome
SOC: system‐organ class
UMC: Uppsala Monitoring Centre
USFDA: United States Food and Drug Administration
UTIs: Urinary tract infections
UTI: urinary tract infection
WHO: World Health Organization
ZPCs: zonal pharmacovigilance centres

## Introduction

The increasing prevalence of penicillin resistance bacteria has resulted in reliance on fluoroquinolones and their widespread use globally (Goldstein and Garabedian‐Ruffalo 2002). Their popularity is also enhanced by their relatively broad spectrum activity, approval in most countries for multiple indications, and high bioavailability (Mehlhorn and Brown 2007).

There are six FDA‐approved fluoroquinolones available in various brands and generic forms (Bradley and Jackson 2011). They include ciprofloxacin, levofloxacin, gatifloxacin, moxifloxacin, ofloxacin, and norfloxacin. These fluoroquinolones are equally approved for use in Nigeria by the National Agency for Food Drug Administration and Control (NAFDAC) and are widely available on the Nigerian market (Tytler et al. 2007). The fluoroquinolones constitute about 16% of the world market for antibiotics and it is projected that as the global demand for antibacterial drugs grow, by 2019, fluoroquinolones use would increase (Drugwatch, 2016). Consequently, fluoroquinolone‐resistant bacteria and reported cases of adverse reactions to this drug class would likely increase (Owens and Ambrose 2005; Oshikoya 2006).

Although, fluoroquinolones have excellent pharmacokinetic properties and are well tolerated by children and adults, there are concerns for their safety. Several adverse reactions have been reported to this class of drugs during clinical trials and post‐marketing surveillance and frequently involve the gastro‐intestinal tract (GIT), musculoskeletal system, central nervous system (CNS), dermatological and the hepatic systems (Tytler et al. 2007; Adefurin et al. 2011; Stahlmann and Lode 2013).

The safety concerns for moxifloxacin and norfloxacin were responsible for their restricted use in Europe (EMEA 2008a,b). In a recent review of the safety data for fluoroquinolones by the FDA, it was reported that oral and injectable fluoroquinolones for systemic use are associated with disabling and potentially permanent, serious adverse effects involving the tendons, muscles, joints, nerves, and central nervous system, occurring singly or together in the same patient (USFDA, 2016). Consequently, the FDA recommended and approved label changes for all systemic fluoroquinolones to reflect this new safety information. The FDA further recommended that fluoroquinolones should be reserved for use in patients who have no alternative treatment options for acute bacterial sinusitis, (ABS), acute bacterial exacerbation of chronic bronchitis (ABECB), and uncomplicated urinary tract infections (UTIs) since the risk of those serious adverse effects appear to generally outweigh the benefits in these patients.

Adverse drug reactions are under‐reported in developing countries (Oshikoya and Awobusuyi 2009) and reporting is now enhanced in Africa by the establishment of National Pharmacovigilance Centres (NPCs) in Nigeria, South Africa, Zimbabwe, Morocco, and Ghana (WHO 2016a) These centers are necessary to determine drug hazard profiles in various countries and to detect rare or previously unknown drug‐related problems. The National Agency for Food and Drug Administration and Control (NAFDAC) in Nigeria regulates and controls the manufacture, importation, exportation, distribution, advertisement, sale and use of food, drugs, cosmetics, chemicals, detergents, medical devices, and packaged water (NAFDAC, 2016). Healthcare providers and patients are encouraged to report ADRs to the NPC located in NAFDAC office. An undocumented search of the NPC local database and VigiFlow^®^ showed that there are over 18,000 reports received and entered into the NPC database from inception to date, of which 11,000 or more had been entered into VigiFlow^®^. Spontaneous reporting of ADRs to the NPC in Nigeria has prompted the timely withdrawal of toxic paracetamol adulterated with diethylene glycol that claimed the lives of some infants and young children in 2008 (Oshikoya 2010; NAFDAC, 2016). It has also led to the ban of dipyrone in 2005 due to the frequent injection abscess and unexplained deaths associated with its use (Fehintola 2005; Cliff‐Eribo et al. 2016).

Post‐marketing safety data on fluoroquinolones may vary from one country to another due to variation in disease and drug utilization patterns, and differences in regulatory policies (EMEA 2008a,b; Bradley and Jackson 2011). The majority of the available safety data for fluoroquinolones are from western world (Owens and Ambrose 2005; Oshikoya 2006; Stahlmann and Lode 2013; USFDA, 2016) and, to date, limited studies have been conducted on their adverse reactions globally (Davey et al. 1991; Norrby 1991; Davey and McDonald 1993; De Sarro and De Sarro 2001; Leone et al. 2003; Jose et al. 2008). This study, therefore, aimed to describe the reports of adverse reactions to fluoroquinolones in the database of the NPC in Nigeria since established over a decade ago.

## Materials and Methods

### Data source

Spontaneous reporting of ADRs is practiced in Nigeria using a standard structured yellow form as recommended by the World Health Organization‐Uppsala Monitoring Centre (WHO‐UMC) in Sweden (Uppsala Monitoring Centre, 2016a). The yellow form captures information about the details of the patient, ADR, suspected drug, concomitant drugs, and the reporter. A duly filled yellow or ADR form is called an individual case study report (ICSR). Healthcare providers, healthcare institutions, marketing authorization holders and patients can send ADR reports to either the NPC, zonal pharmacovigilance centers (ZPCs) or NAFDAC state offices nationwide. Causality assessment for each ADR and the suspected drug(s) is performed by pharmacovigilance experts and staff of the NPC using the WHO‐UMC causality assessment system (Uppsala Monitoring Centre, 2016b). Complex cases of ADRs are assessed for causality by the National Drug Safety Advisory Committee comprising of clinical pharmacologists, toxicologists, clinical pharmacists and clinicians, with expertise in pharmacovigilance. The ADRs are coded on the basis of the WHO Adverse Reaction Terminology (WHO‐ART) (Uppsala Monitoring Centre, 2016c). All reports judged to be ADRs at the NPC are sent to UMC which receives anonymized reports from over 124 member countries. These are then entered into the WHO Global Individual Case Safety Report database, VigiBase^®^.

### Data abstraction

The ICSR of patients who experienced adverse reaction(s) to fluoroquinolones between September 2004 and August 2016 were sourced from the NPC in Nigeria and reviewed to obtain the following information: patient's characteristics (age, gender, and indication(s) for fluoroquinolone use), suspected fluoroquinolone (type and route of administration) and ADR (type, onset, causality, and outcome), and type of reporter. It is probable that the suspected fluoroquinolones for the same ADR will vary from case to case. The period between intake of fluoroquinolone and the onset of clinical symptoms manifesting as an ADR is the onset time (Riedl and Casillas 2003). Outcome of the ADR refers to the extent of resolution of the signs and symptoms of ADR and its sequalae as at the time the report was submitted to NPC. The outcomes were categorized as full recovery without any sequelae, recovery with sequelae, ongoing if patient was still experiencing the problems, or death.

### ADR rating

The causalities were categorized as certain, probable/likely, possible, unlikely, conditional/unclassified, or unassessible/unclassifiable (Uppsala Monitoring Centre, 2016b). For patients who experienced multiple ADRs to a single fluoroquinolone, severity was assessed for the entire ADRs as a single entity. We assessed ADR severity using modified Hartwig and Siegel assessment scale as described by Srinivasan and Ramya (2011).The ADRs were graded as mild (transient or mild discomfort lasting less than 48 h; no antidote, medical intervention or therapy required; no hospitalization is required), moderate (mild ADR requiring an antidote or other treatment; mild ADR requiring at least a day hospitalization; or mild ADR requiring a short hospital admission), severe (moderate ADR requiring intensive medical care; any level of ADR that caused permanent harm to the patient; or any ADR that directly or indirectly caused death of a patient) (Srinivasan and Ramya 2011). Serious ADRs are those that resulted in death, persistent or significant disability/incapacity, were life‐threatening, required hospitalization or prolongation of existing hospitalization (Jose et al. 2008).

### Ethical considerations

The NAFDAC Director General approved the study.

### Analysis

We analyzed the data with IBM SPSS statistics software, Version 21.0. Armonk, NY, USA: IBM. Corp (Released 2012). Descriptive statistics was used to summarize patients' and ADR characteristics. The annual number of ADR reports, patients' age distribution, numbers of ADRs per patient, and serious ADRs reported were presented pictorially. The association between characteristics of the patients, fluoroquinolone, and ADRs was tested using Pearson Chi‐square. The association was considered to be statistically significant when *P *<* *0.05.

## Results

### Number of cases of ADRs reported and the types of fluoroquinolones involved

**Figure 1:**
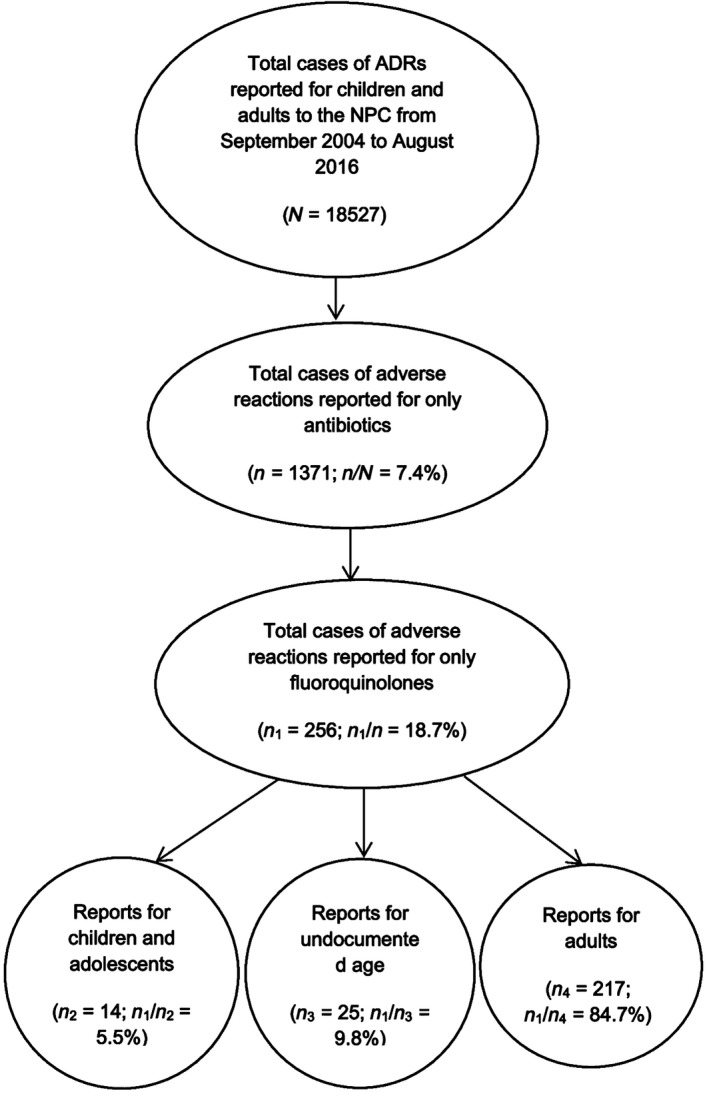
Flowchart of the filter of ADR reports to yield adverse reactions to fluoroquinolones in the NPC database from September 2004 to August 2016.

**Figure 2:**
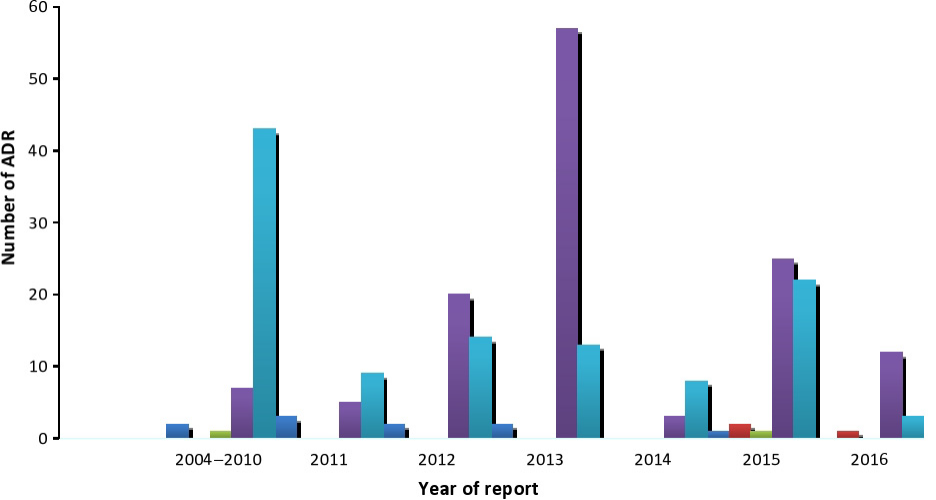
Number of ADR reports to fluoroquinolones by year.

Over the 12‐year period, 256 cases of adverse reactions due to fluoroquinolones were reported (Fig. 1). Although the reports received from 2004 to 2010 were not segregated, as the year of reporting was not captured previously, the annual number of reports for fluoroquinolones, from 2011 and beyond, ranged from 11 to 72 (Fig. 2).

From inception to 2010, the reports due to ciprofloxacin were the highest. In the subsequent years, reports due to ciprofloxacin fluctuated between 3 and 14 per annum and peaked in 2015. Reports due to levofloxacin increased gradually in 2011 to the peak in 2013 and declined drastically to eight in 2014. Overall, the ADR reports were due to norfloxacin (2; 0.8%), moxifloxacin (3; 1.2%), ofloxacin (10; 3.9%), ciprofloxacin (112; 43.8%), and levofloxacin (129; 50.4%).

### Demographics of patients and indications for fluoroquinolones

**Figure 3:**
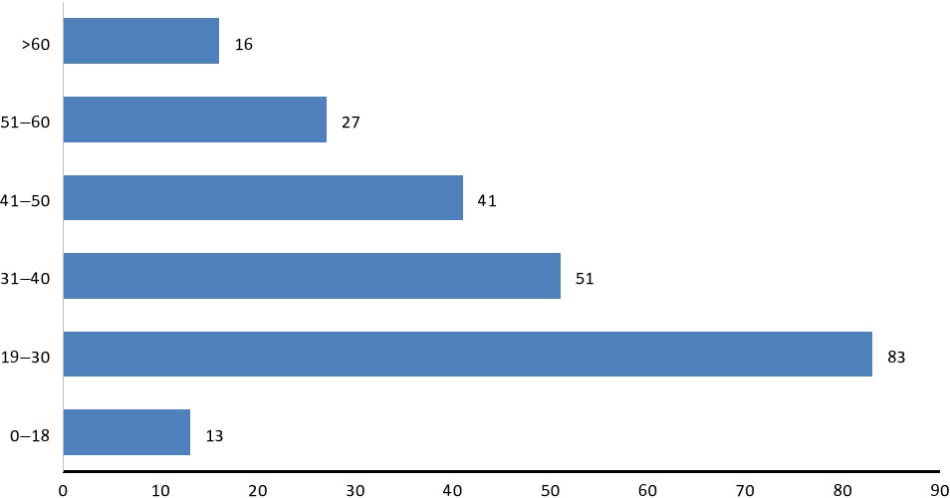
Age distribution of the 231 patients whose ages were reported along with the adverse reactions.

Of the 256 patients reported to experience adverse reactions to fluoroquinolones, 138 (53.9%) were males, 113(44.1%) were females, while the gender was not reported for 5(2.0%) patients. The age was not reported for 25(9.8%) patients (Fig. 1). The mean and standard deviation for the age of the remaining 231 patients was 37.0 ± 14.4 years. Their age distribution is presented in Figure 3. Young adults (19–30 years old) were the most affected, while children and adolescents, and the elderly were the least affected by the ADRs.

Table 1 shows the indications for using fluoroquinolones among the patients. The indications were not reported for 34 (13.3%) patients. Tuberculosis, probable sepsis, and enteric fever were the three most common illnesses necessitating the use of fluoroquinolones.

**Table 1:** Reported indications for using fluoroquinolones among the 256 patients who experienced adverse reactions

Indications for fluoroquinolone use by the patients	Frequency *n* (%)
Tuberculosis	88 (34.4)
Probable sepsis	45 (17.6)
Enteric fever	24 (9.4)
Postoperatively	8 (3.1)
Urinary tract infection	8 (3.1)
Malaria	5 (2.0)
Pneumonia	5 (2.0)
Gastroenteritis	4 (1.6)
Upper respiratory tract infection	4 (1.6)
Pelvic inflammatory disease	2 (0.8)
Osteomyelitis	2 (0.8)
Dermatosis	2 (0.8)
Others^a^	21 (8.2)
Double indications^b^	4 (1.6)
Indications not reported	34 (13.3)
Total	256 (100.0)

a Refer to diagnosis (nephritis, hip pain, swollen neck, trauma, thyrotoxicosis, nephrotic syndrome, osteoarthritis, appendicitis, lymphadenitis, septic wound, tetanus, pruritus, conjunctivitis, ascites, blurry vision, asthma, diabetes mellitus, and acute otitis media) reported in only one patient. Double indications.

b Refer to two diagnoses (malaria and appendicitis, malaria and postoperatively, gastroenteritis and postoperatively, and enteric fever and malaria) made in one patient.

The route of administration of the fluoroquinolones was reported for 219 (85.5%) patients. Most of the patients (184; 84.0%) received the drug via the oral route. Thirty (13.7%) patients received the drugs via the intravenous route, while three received them initially via intravenous route, followed by oral route.

### Type of reporter

Of the 256 reporters, details for only 45 (17.6%) were provided during reporting. These reporters were pharmacists (16; 35.5%), medical students (15; 33.3%), nurses (5; 11.1%), medical laboratory technologists (2; 4.4%), and physiotherapist (1; 2.2%).

### Adverse reactions to fluoroquinolones

#### Total ADR and the number experienced per patient

**Figure 4:**
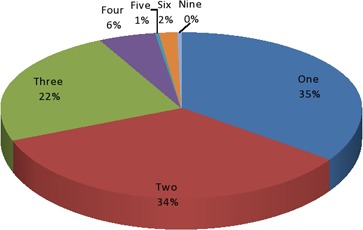
Number of adverse events to fluoroquinolones reported for the 256 patients.

A total of 540 ADRs was experienced by the 256 patients. The numbers of ADRs experienced per patient are presented in Figure 4. Only 91(35%) patients experienced a single ADR.

The number of ADRs experienced per patient was statistically significantly associated with the patients' age (*P* = 0.001) and the types of fluoroquinolone implicated in the ADRs (*P* = 0.038), but not significantly associated with the indications for the drugs (*P* = 0.885), gender of the patients (*P* = 0.760), and route of administration of the drug (*P* = 0.860). Majority of the patients experienced 2–9 ADRs.

#### Onset of ADRs

Time to onset of ADR was not reported for over half of the patients (146, 57.0%). Among those with onset time reported, 15(13.6%) experienced the ADR in <24 h. Eighty‐two (74.5%) of the patients experienced ADRs in 1–10 days after fluoroquinolone exposure, while the remaining patients (13; 11.8%) had a late onset (11–30 days) ADRs. Time to onset of ADR was statistically significantly associated with the indications (*P* < 0.001), types (*P* < 0.001), and route of administration (*P* < 0.001) of the fluoroquinolones. The onset time was also statistically significantly associated with the age (*P* = 0.001) but not with the gender (*P* = 0.697) of the patients.

#### System‐organ class affected by ADR and the implicated fluoroquinolones

The system‐organ class and specific ADR, as well as the culprit fluoroquinolones, are presented in Table 2. Based on the WHO‐SOC for categorizing ADRs, neurological disorders (121; 22.4%), gastrointestinal disorders (118; 21.9%), and skin and appendages disorders (116; 21.5%) were the most reported ADRs. The specific ADRs most frequently reported were pruritus, abdominal pain, vomiting, and skin rash.

**Table 2:** Adverse reactions to fluoroquinolones based on system‐organ classes (SOC) and their codes using WHO‐ART guide

System‐organ class affected and the specific suspected ADRs in preferred term	Fluoroquinolones implicated in the adverse reactions					Total number of ADRs, *N* (%)
	Ofloxacin (*n*_*1*_)	Moxifloxacin (*n*_*2*_)	Norfloxacin (*n*_*3*_)	Ciprofloxacin (*n*_*4*_)	Levofloxacin (*n*_*5*_)	
Skin and appendages disorders (SOC code 0100)	5	1	–	75	35	116 (21.5)
Pruritus	1	1	–	26	13	41 (7.6)
Skin rash	–	–	–	18	9	27 (5.0)
Angioedema	–	–	–	6	8	14 (2.6)
Urticaria	–	–	–	3	3	6 (1.1)
Hyperpigmentation	–	–	–	4	–	4 (0.7)
Wheals	2	–	–	2	–	4 (0.7)
Stevens‐Johnson syndrome	1	–	–	3	–	4 (0.7)
Blisters	–	–	–	2	–	2 (0.4)
Bullous eruption	–	–	–	3	–	3 (0.6)
Facial puffiness	–	–	–	2	–	2 (0.4)
Lip discolouration	–	–	–	2	–	2 (0.4)
Burning sensation to the lips	–	–	–	1	–	1 (0.2)
Dry lips	–	–	–	1	–	1 (0.2)
Dry scaly skin	–	–	–	–	1	1 (0.2)
Erythema nodosum	–	–	–	–	1	1 (0.2)
Photosensitivity	–	–	–	1	–	1 (0.2)
Skin exfoliation	1	–	–	–	–	1 (0.2)
Toxic epidermal necrolysis	–	–	–	1	–	1 (0.2)
Musculoskeletal disorders (SOC code 0200)	1	–	–	10	27	38 (7.0)
Arthralgia	–	–	–	–	13	13 (2.4)
Joint pain	–	–	–	–	5	5 (0.9)
Waist pain	–	–	–	–	3	3 (0.6)
Muscle spasm	–	–	–	3	–	3 (0.6)
Ankle pain	–	–	–	–	2	2 (0.4)
Myalgia	1	–	–	1	–	2 (0.4)
Swollen ankle	–	–	–	–	2	2 (0.4)
Swollen hand	–	–	–	2	–	2 (0.4)
Arm swelling	–	–	–	1	–	1 (0.2)
Arm pain	–	–	–	1	–	1 (0.2)
Feet stiffness	–	–	–	–	1	1 (0.2)
Hand stiffness	–	–	–	–	1	1 (0.2)
Neck pain	–	–	–	1	–	1 (0.2)
Tendinitis	–	–	–	1	–	1 (0.2)
Neurological disorders (SOC code 0400)	14	–	4	44	59	121 (22.4)
Headache	2	–	1	10	10	23 (4.3)
Insomnia	1	–	–	2	19	22 (4.1)
Dizziness	3	–	–	9	6	18 (3.3)
Peripheral	1	–	–	5	6	12 (2.2)
Restlessness	2	–	–	6	3	11 (2.0)
Confusion	1	–	–	2	2	5 (0.9)
Anorexia	–	–	–	–	3	3 (0.6)
Convulsion	–	–	–	–	3	3 (0.6)
Drowsiness	–	–	2	1	–	3 (0.6)
Numbness of the feet	–	–	–	3	–	3 (0.6)
Tremor	–	–	–	1	2	3 (0.6)
Fainting spells	–	–	–	1	1	2 (0.4)
Oculogyric crisis	–	–	–	1	1	2 (0.4)
Agitation	–	–	–	1	–	1 (0.2)
Burning sensation to the eyes	1	–	–	–	–	1 (0.2)
Dysphagia	1	–	–	–	–	1 (0.2)
Dysarthria	1	–	–	–	–	1 (0.2)
Dysphonia	–	–	–	1	–	1 (0.2)
Hypotension	1	–	–	–	–	1 (0.2)
Heaviness of the head	–	–	–	1	–	1 (0.2)
Internal heat	–	–	–	–	1	1 (0.2)
Lightheadedness	–	–	–	–	1	1 (0.2)
Loss of consciousness	–	–	1	–	–	1 (0.2)
Thirst	–	–	–	–	1	1 (0.2)
Vision disorders (SOC code 0431)	–	–	–	2	1	3 (0.6)
Blindness	–	–	–	1	–	1 (0.2)
Blurred vision	–	–	–	1	–	1 (0.2)
Redness of the eyes	–	–	–	–	1	1 (0.2)
Hearing, vestibular and special senses disorders (SOC code 0432)	–	4	––	4	20	28 (5.2)
Tinnitus	–	2	–	–	9	11 (2.0)
Vertigo	–	–	–	2	8	10 (1.9)
Deafness	–	2	–	2	3	7 (1.3)
Psychiatric disorders (SOC code 0500)	–	–	–	–	16	16 (3.0)
Psychosis	–	–	–	–	10	10 (1.9)
Depression	–	–	–	–	6	6 (1.1)
Gastrointestinal disorders (SOC code 0600)	3	–	1	56	58	118 (21.9)
Abdominal pain	1	–	–	9	24	34 (6.3)
Vomiting	–	–	–	17	17	34 (6.3)
Diarrhea	–	–	–	7	6	13 (2.4)
Abdominal discomfort	–	–	1	6	4	11 (2.0)
Nausea	1	–	–	7	2	10 (1.9)
Abdominal bloat	–	–	–	2	–	2 (0.4)
Bitter taste	–	–	–	2	–	2 (0.4)
Coated tongue	1	–	–	–	1	2 (0.4)
Bloody stool	–	–	–	–	1	1 (0.2)
Constipation	–	–	–	1	–	1 (0.2)
Dry mouth	–	–	–	–	1	1 (0.2)
Metallic taste	–	–	–	–	1	1 (0.2)
Mouth sore	–	–	–	1	–	1 (0.2)
Odynophagia	–	–	–	1	–	1 (0.2)
Stomatitis	–	–	–	1	–	1 (0.2)
Swollen gum	–	–	–	1	–	1 (0.2)
Swollen jaw	–	–	–	–	1	1 (0.2)
Throat irritation	–	–	–	1	–	1 (0.2)
Liver and biliary disorders (SOC code 0700)	–	–		1	–	1 (0.2)
Jaundice	–	–	–	1	–	1 (0.2)
Endocrine disorders (SOC code 0900)	–	–	–	6	–	6 (1.1)
Hot flushes	–	–	–	3	–	3 (0.6)
Milky nipple discharge	–	–	–	2	–	2 (0.4)
Breast pain	–	–	–	1	–	1 (0.2)
Cardiovascular disorders (SOC code 1000)	1	–	–	1	6	8 (1.5)
Palpitation	–	–	–	1	4	5 (0.9)
Elevated blood pressure	1	–	–	–	1	2 (0.4)
Tachycardia	–	–	–	–	1	1 (0.2)
Respiratory disorders (SOC code 1100)	2	–	–	6	6	14 (2.6)
Breathing difficulty	1	–	–	5	1	7 (1.3)
Chest pain	1	–	–	1	4	6 (1.1)
Cough	–	–	–	–	1	1 (0.2)
Urinary tract disorders (SOC code 1300)	–	–	–	3	3	6 (1.1)
Frequent micturition	–	–	–	1	1	2 (0.4)
Dysuria	–	–	–	1	–	1 (0.2)
Hematuria	–	–	–	1	–	1 (0.2)
Kidney failure	–	–	–	–	1	1 (0.2)
Reduced micturition	–	–	–	–	1	1 (0.2)
Reproductive disorders (SOC code 1400)	–	–	–	1	4	5 (0.9)
Vaginal discharge	–	–	–	1	3	4 (0.7)
Groin pain	–	–	–	–	1	1 (0.2)
Body as a whole‐ general disorders (SOC code 1810)	5	–	–	24	28	57 (10.6)
Weakness	4	–	–	6	7	17 (3.1)
Body pain	1	–	–	1	11	13 (2.4)
Fever	–	–	–	8	–	8 (1.5)
Anaphylaxis	–	–	–	–	4	4 (0.7)
Chills and rigor	–	–	–	4	–	4 (0.7)
Sweating	–	–	–	1	2	3 (0.6)
Fatigue	–	–	–	–	2	2 (0.4)
Electrolyte imbalance	–	–	–	1	1	2 (0.4)
Hypothermia	–	–	–	1	–	1 (0.2)
Edema	–	–	–	–	1	1 (0.2)
Tiredness	–	–	–	1	–	1 (0.2)
Weight loss	–	–	–	1	–	1 (0.2)
Application site disorders (SOC code 1820)	–	–	–	–	3	3 (0.6)
Atrophy of injection site	–	–	–	–	3	3 (0.6)
Total	31	5	5	233	266	540 (100.0)

#### Outcomes from ADRs and their severity

**Figure 5:**
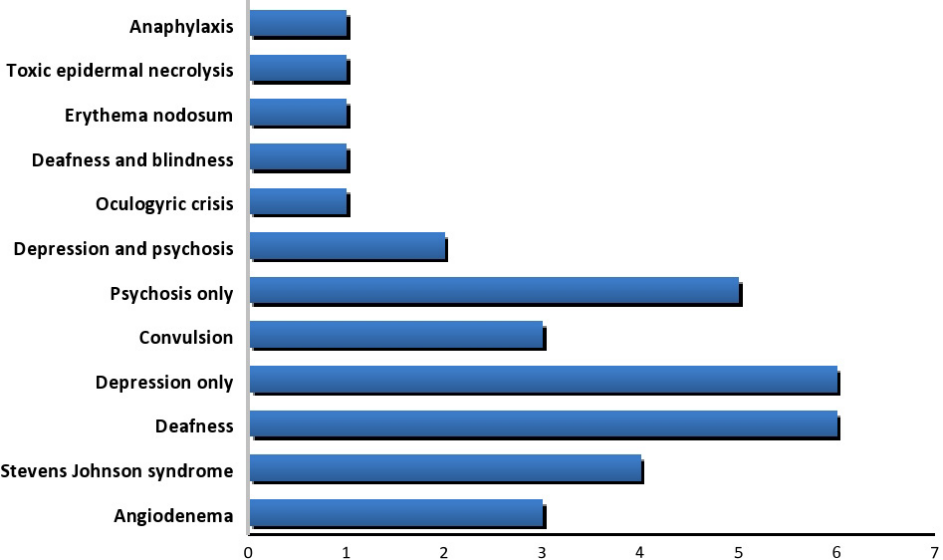
Serious ADRs reported for fluoroquinolones.

The outcomes from ADRs were not reported for 110(43.0%) patients. However, 135(52.7%) patients recovered fully without sequelae, 5(2.0%) recovered with morbidity, while 6(2.3%) were still experiencing the ADRs as at the time of reporting. Among those patients with reported outcomes, severity of their ADRs was mild (72; 49.3%), moderate (64; 43.8%), and severe (10; 6.9%). A total of 34(6.4%) patients experienced serious ADRs, including six patients who experienced depression or deafness, and one patient who died of Stevens Johnson syndrome (SJS) (Fig. 5). The outcomes from the ADRs were statistically significantly associated with the types of fluoroquinolone implicated in the ADRs (*P* < 0.001) and the route of administration of the drug (*P* < 0.001). The association was, however, not statistically significant with the indications for fluoroquinolone (*P* = 0.210), and age (*P* = 0.980) and gender (*P* = 0.101) of the patients. The ADR severity was statistically significantly associated with the fluoroquinolone types (*P* = 0.005) but not with their indications (*P* = 0.628), route of administration (*P* = 0.520), age (*P* = 0.139), or gender (*P* = 0.358) of the patients.

#### ADR causality

Based on the WHO‐UMC causality assessment, the ADRs were certain (90; 16.7%), probable/likely (107; 19.8%), possible (291; 53.9%), unlikely (27; 5.0%), and not assessable/unclassifiable (25; 4.6%).

## Discussion

Since the existence of NPC in Nigeria to date, a total of 18,527 ICSRs was received; of which 1.4% was due to fluoroquinolones. This was lower than the 28,609 reports from the National Adverse Event Monitoring Centre in South Africa entered into the VigiBase^®^ between 1992 and 2015 (Ampadu et al. 2016), and far lower than the 692,904 reports received in 2010 by the National Centre for ADR Monitoring in China (Biswas 2013). Although, pharmacovigilance is relatively new in Nigeria compared to South Africa and China (Biswas 2013; Ampadu et al. 2016), under‐reporting may have accounted for the low ADR rate per annum attributed to fluoroquinolones in this study. ADR under‐reporting is a major challenge globally but the extent varies from one country to another (Vallano et al. 2005). Previous studies have documented that only a small percentage of the total number of occurring ADRs is reported (Rawlins 1988). Healthcare professionals, comprising mainly nurses, pharmacists and physicians, are involved in ADR reporting in Nigeria, yet many lack awareness of the significance of pharmacovigilance (Oshikoya and Awobusuyi 2009; Fadare et al. 2011), further contributing to ADR under‐reporting. The available information about the type of ADR reporter was very low (17.6%) in our study but comparable to the 15% reported in a study capturing the total ADRs reported to the Saudi Adverse Event Reporting System (SAERS) between December 2009 and June 2012 (Alshammari et al. 2015). Such information is necessary to identify the target group needing more training in pharmacovigilance as a means of improving ADR reporting.

The reports of ADRs to fluoroquinolones received by the NPC were mainly for patients treated with levofloxacin and ciprofloxacin. Both drugs were used mainly for MDR‐TB and probable sepsis. High‐dose levofloxacin and moxifloxacin are recommended by the WHO guidelines for MDR‐TB, similar to the treatment guidelines in Nigeria (Federal Ministry of Health Nigeria, 2016, WHO 2016b). Nigeria is ranked 13th among the 22 high burden countries for TB in the world and the third in Africa (WHO, 2014). These account for the high burden of MDR‐TB in Nigeria; potentially enhanced by a greater prevalence of HIV (Uzoewulu et al. 2014). Consequently, a high use of fluoroquinolones observed among TB patients in this study is expected. The donor agencies and voluntary organizations supporting Nigerian Government in fighting against TB encouraged the focal persons at each treatment center to report all adverse reactions associated with anti‐tuberculous drugs, including fluoroquinolones (Federal Ministry of Health Nigeria, 2016). This emphasizes the significance of education and awareness on ADR reporting. Pharmacovigilance education and training for healthcare professionals have been identified as key to improving ADR reporting in Nigeria (Oshikoya and Awobusuyi 2009; Fadare et al. 2011). This approach was implemented in Spain, India, and Malaysia, and the reporting rate improved substantially (Elkalmi et al. 2011; Bisht et al. 2014; Lopez‐Gonzalez et al. 2015).

A substantial number of the conditions necessitating the use of fluoroquinolone was either inappropriate, unnecessary, or could have been treated with alternative drugs. Several studies have demonstrated that fluoroquinolones are often used inappropriately (Dydek et al. 1992; Belliveau et al. 2000; Lautenbach et al. 2003; Mean et al. 2006; Elkalmi et al. 2011). Lautenbach et al. (2003) found that 81% of fluoroquinolones prescribed in two academic emergency departments were inappropriate since they deviated from the institutional guidelines. In two different French studies, 51–55% of the fluoroquinolone regimens were inappropriate or unnecessary, based on hospitals' prescription guidelines (Dydek et al. 1992; Mean et al. 2006). The misuse was associated with misdiagnosis of pulmonary infections, urinary tract infections, fever of unknown origin, and abdominal infections, similar to the spectrum of infections reported in our study (Mean et al. 2006; Pulcini et al. 2007). In another study involving a tertiary care hospital in Ohio, 30% of the antimicrobial prescriptions for in‐patients were unnecessary, with ciprofloxacin being the agent most often prescribed unnecessarily (Belliveau et al. 2000).

Appropriate use of fluoroquinolones would have substantially reduced the number of ADRs reported in our study. This may suggest a lack of adherence to the standard treatment guidelines in Nigeria or a lack of explicit information on how best to treat those conditions inappropriately treated with fluoroquinolones (Federal Ministry of Health, WHO, EC and DFID, 2008) Non‐adherence to hospital treatment guidelines were among the major factors contributing to unnecessary prescriptions of fluoroquinolones in two different French hospitals (Mean et al. 2006; Pulcini et al. 2007). These findings should encourage research groups working on rational use of antibiotics, health ministry at the state and federal levels, and health institutions in Nigeria to organize frequent audit of antibiotics utilization. Physicians and other healthcare professionals would need to be educated on the significance of adherence to guidelines such as preventing emergence of resistant bacteria to antibiotics and reducing cost of treatment to patients. Formal and informal antibiotic stewardship education have been key to sustainable behavioral changes and social norms, reorientation of physicians and other healthcare professionals toward rational use of antibiotics in low‐ and middle‐income countries (WHO, 2002; Sumpradit et al. 2012). In addition, educational intervention was successful to optimizing fluoroquinolone use in a French public hospital (Lacombe et al. 2005).

Children constituted 5.5% of the cases of ADRs reported. A single‐center study of ADRs to antibiotics in India showed that 20.4% of the victims were children (Shamna et al. 2014). Our finding may, therefore, reflect a reduced prescribing pattern of fluoroquinolones for children based on the existing guidelines (Federal Ministry of Health 2008), heightened fear of adverse toxicities when used as off‐label (Kuriakose 2016), or ADR under‐reporting in children (Cliff‐Eribo et al. 2016).

ADRs related to neurological disorders, gastrointestinal disorders, and skin and appendages disorders were reported more than any other system‐organ class. This trend is similar to the previous ADRs reported to fluoroquinolones (Halkin 1988; Fish 2001). However, earlier studies had reported more gastrointestinal than neurological ADRs (Halkin 1988; De Sarro and De Sarro 2001; Fish 2001; Owens and Ambrose 2005), which is contrasting to our findings of more neurological than gastrointestinal ADRs. Our findings are also contrasting to the early post‐marketing surveillance of ciprofloxacin alone, as well as those of ciprofloxacin, norfloxacin, and ofloxacin evaluated 2 years later, which showed a preponderance of ADR reporting to skin and appendages, followed by neurological and gastrointestinal disorders (Davey et al. 1991; Davey and McDonald 1993). Previous studies comparing ADRs attributed to fluoroquinolones and other widely used antibiotic classes (cephalosporins, penicillins, and macrolides) reported gastrointestinal disorders, followed by skin and appendages disorders to be more common with other antibiotics (Norrby 1991). Another study involving ADRs to all classes of antibiotics reported to a peripheral pharmacovigilance center in India, over a 3‐year period, showed that dermatological (47.4%) and gastrointestinal (39.3%) ADRs were the most prevalent. Cephalosporins (35.7%), followed by fluoroquinolones (11.3%), were the most implicated classes of antibiotics in the comparative study (Richa et al. 2015). Only 5.4% of the ADRs reported in the Indian study were related to neurological disorders. The higher number of cases of adverse reactions to fluoroquinolones reported in our study, compared to the Italian (Leone et al. 2003) and Indian (Jose et al. 2008; Richa et al. 2015) studies, may account for our observed high neurological ADRs. This may, otherwise, suggest an increased trend for neurological ADRs which would require an intense pharmacovigilance. The increased reports of neurological ADRs to fluoroquinolones and the associated serious effects were the main reasons for recent change in labeling of this class of drugs and revision of the boxed warning by the FDA (USFDA, 2016). This also contributes to the restricted use of fluoroquinolones in Europe (EMEA 2008a,b).

Pruritus, skin rashes, abdominal pains, vomiting, headache, and insomnia were the most common specific ADRs reported to fluoroquinolones. This is in keeping with the previous hospital‐based pharmacovigilance study in India (Jose et al. 2008) and another pharmacovigilance study involving three Italian regions (Leone et al. 2003). In contrast, a study in Sweden has reported nausea, diarrhea, dizziness, somnolence, dyspepsia, and flatulence to be the most common ADRs to fluoroquinolones (Norrby 1991). In phase 3 studies of moxifloxacin and levofloxacin, diarrhea, abnormal liver function test, nausea, injection site reaction, and vomiting were the common ADRs reported (Owens and Ambrose 2005). In comparison to a study involving a peripheral pharmacovigilance center in India, allergic reactions, diarrhea, rash, and constipation were the most common ADRs reported to fluoroquinolones, while skin rashes, diarrhea, and gastritis were the ADRs most reported to other widely used antibiotics such as cephalosporins, macrolides, and penicillins (Richa et al. 2015).

Most of the ADRs reported in this study were adjudged probable (19.8%) or possible (53.9%). This is in contrast to all the 80 ADRs considered to be probable (52.5%) or possible (47.5%) in a study that evaluated ADRs to fluoroquinolones reported to a peripheral pharmacovigilance center in India (Jose et al. 2008). However, an Italian study involving three regional pharmacovigilance centers reported 76% probable and 22% possible ADRs to fluoroquinolones (Leone et al. 2003). In comparison to other widely used antibiotics, ADRs were probable (71.4%) or possible (18.4%) (Shamna et al. 2014). Like the Italian study, we assessed causality based on WHO‐UMC criteria, while the other comparative studies used the Naranjo's scale. There have been inconsistent reports on the level of agreement between these causality assessment tools (Lei et al. 2007; Rehan et al. 2007; Belhekar et al. 2014). While some studies reported a very high disagreement (Belhekar et al. 2014), others reported a low disagreement (Rehan et al. 2007; Belhekar et al. 2014). These discrepancies underscore the need for a common causality assessment tool in pharmacovigilance studies.

Thirty‐four (6.4%) serious ADRs and one death were recorded which is lower than 42.8% reported for serious ADRs to fluoroquinolones in three regional pharmacovigilance centers in Italy (Leone et al. 2003). Deafness (6), depression (6), psychosis (5), and SJS (4) were the most frequently reported serious ADRs to fluoroquinolones in our study, which are in contrast to tendonitis/tendon rupture (16), hallucinations (15), angioedema (11) and photosensitivity reactions (9) reported in a previous Italian study (Leone et al. 2003). The death recorded in our study was due to SJS, similar to the single case reported in a peripheral pharmacovigilance center in India (Jose et al. 2008). However, no fatality was reported in the Italian study (Leone et al. 2003). One patient each reported rare adverse reactions to fluoroquinolones. While there are reports of fluoroquinolone‐induced hepatotoxicity (Adikwu and Deo 2012), asymptomatic hematuria (Garlando et al. 1985), and acute kidney injuries (Bird et al. 2013) in the literature, other rare ADRs have never been reported and the mechanism involved cannot be explained. It is hoped that attention would be paid to these rare ADRs in the future monitoring of fluoroquinolones in Nigeria.

Our study is characterized by several limitations. A major limitation is that important details of some of the patients such as age, gender, and indications for fluoroquinolone use were not reported by some reporters. Other important details are time‐of‐onset and outcome of the ADRs which were not reported for some patients. Physicians in Nigeria have indicated that lack of time to fill an ADR reporting form and concern for an ADR reporting would generate an extra work as two important factors discouraging them from reporting ADRs (Oshikoya and Awobusuyi 2009). Our findings are in keeping with the previous studies reporting incompleteness of ADR forms submitted to pharmacovigilance centers in Mexico (Sánchez‐Sánchez et al. 2012) and Saudi Arabia (Alshammari et al. 2015), and those submitted to a pharmaceutical company in Italy (Impicciatore and Mucci 2010). Patients' demographics were filled out in 38.3% of the reports submitted in Saudi Arabia between 2009 and 2012; gender and age being the most frequently omitted details (Alshammari et al. 2015). Formulation (21%) and route of administration (37%) of the suspected drugs, seriousness (52%) and outcome (75%) of the ADR details were provided among 100 reports received by Pfizer Medical in Milan, between 2005 and 2008 (Impicciatore and Mucci 2010). Incomplete ADR information may limit the effectiveness and full potential of analysis of such reports. The NPC local database is used to store all reports received irrespective of their completeness status. Since the NPC has no rejection policy for incomplete suspected ADR reports, timely evaluation of the received suspected ADR reports should be considered as a means of early identification of incomplete reports. Reporters should be reached via repeated email, phone calls or visits, and encouraged with incentives to providing missing details from the reports. Continuous pharmacovigilance education for healthcare professionals should emphasize the importance of fully completing the ADR report forms when reporting.

There is a potential risk of not being able to adequately assess the ADRs due to confounding factors such as concomitant medications and illnesses. This bias may have caused over‐or under‐estimation of ADRs in our study. Causality assessment would have been more robust if dechallenge or rechallenge was performed for the reported ADRs and if laboratory data serving as early signals of ADRs were available. However, outcome of these procedures, if performed, are rarely included in spontaneous ADR reports (Jose et al. 2008).

## Conclusions

Fluoroquinolones have contributed to a small but significant proportion of ADRs spontaneously reported to the NPC in Nigeria. Given the global increase in the use of fluoroquinolones and the low report rate observed, when compared to other antimicrobial agents, efforts should be intensified to increase public awareness on ADR and to encourage reporting in Nigeria. Such reporting should be encouraged to be as complete as possible in order to improve the robustness of national ADR data for future analysis. Ciprofloxacin and levofloxacin were the two most culpable fluoroquinolones due to their inappropriate use or increased use in MDR‐TB treatment. The pattern of ADRs and the system‐organ affected show a preponderance of neurological disorders. The reports of rare adverse reactions underscore the need for a more intense post‐marketing surveillance of fluoroquinolones. A national policy on rational use of fluoroquinolones is therefore suggested as a means of minimizing adverse reactions to this drug class.

## Authors Contributions

Conception and design of the study: IAO, KAO and AOA. Data abstraction: KAO, AI, YO, and CS. Data analysis: KAO and IAO. Writing the manuscript: IAO, KAO, AOA, CS, OA, AI, and YO.

## Disclosure

The authors have declared that no competing interests exist.
